# Revisiting the circulation time of *Plasmodium falciparum *gametocytes: molecular detection methods to estimate the duration of gametocyte carriage and the effect of gametocytocidal drugs

**DOI:** 10.1186/1475-2875-9-136

**Published:** 2010-05-24

**Authors:** Teun Bousema, Lucy Okell, Seif Shekalaghe, Jamie T Griffin, Sabah Omar, Patrick Sawa, Colin Sutherland, Robert Sauerwein, Azra C Ghani, Chris Drakeley

**Affiliations:** 1 Department of Infectious & Tropical Diseases, London School of Hygiene & Tropical Medicine, London, UK; 2 Department of Medical Microbiology, Radboud University Nijmegen Medical Centre, Nijmegen, The Netherlands; 3 MRC Centre for Outbreak Analysis & Modelling, Department of Infectious Disease Epidemiology, Imperial College London, London, UK; 4 Kilimanjaro Clinical Research Institute, Moshi, Tanzania; 5 Kenya Medical Research Institute, Nairobi, Kenya; 6 International Centre of Insect Physiology and Ecology, Mbita, Kenya

## Abstract

**Background:**

There is renewed acknowledgement that targeting gametocytes is essential for malaria control and elimination efforts. Simple mathematical models were fitted to data from clinical trials in order to determine the mean gametocyte circulation time and duration of gametocyte carriage in treated malaria patients.

**Methods:**

Data were used from clinical trials from East Africa. The first trial compared non-artemisinin combination therapy (non-ACT: sulphadoxine-pyrimethamine (SP) plus amodiaquine) and artemisinin-based combination therapy (ACT: SP plus artesunate (AS) or artemether-lumefantrine). The second trial compared ACT (SP+AS) with ACT in combination with a single dose of primaquine (ACT-PQ: SP+AS+PQ). Mature gametocytes were quantified in peripheral blood samples by nucleic acid sequence based amplification. A simple deterministic compartmental model was fitted to gametocyte densities to estimate the circulation time per gametocyte; a similar model was fitted to gametocyte prevalences to estimate the duration of gametocyte carriage after efficacious treatment.

**Results:**

The mean circulation time of gametocytes was 4.6-6.5 days. After non-ACT treatment, patients were estimated to carry gametocytes for an average of 55 days (95% CI 28.7 - 107.7). ACT reduced the duration of gametocyte carriage fourfold to 13.4 days (95% CI 10.2-17.5). Addition of PQ to ACT resulted in a further fourfold reduction of the duration of gametocyte carriage.

**Conclusions:**

These findings confirm previous estimates of the circulation time of gametocytes, but indicate a much longer duration of (low density) gametocyte carriage after apparently successful clearance of asexual parasites. ACT shortened the period of gametocyte carriage considerably, and had the most pronounced effect on mature gametocytes when combined with PQ.

## Background

The transmission of malaria depends on the presence of mature sexual stage parasites, gametocytes, in the human peripheral blood. Once ingested by a mosquito taking a blood meal, gametocytes develop through different mosquito-specific stages that ultimately render the mosquito infectious to humans. There is renewed acknowledgement that targeting gametocytes, either alone or as part of integrated control programmes, is essential for malaria control and elimination efforts [1-4].

*Plasmodium falciparum *gametocytes are relatively insensitive to many anti-malarials [5] and circulate for a longer period of time than gametocytes of other malaria species [6,7]. The mean circulation time per gametocyte has been estimated at 3.4 [8], but also at 6.4 days [9].

These mean values do not reflect the broad range in the time that a single person carries gametocytes. Following efficacious clearance of asexual parasites, the source of gametocyte production, the duration of gametocyte carriage is determined by the maximum duration of gametocyte sequestration and the maximum circulation time following their release into the bloodstream. Maximum gametocyte sequestration time was previously estimated at 12 days; maximum circulation time at 22.2 days [9]. As a consequence, gametocyte carriage in individual patients may be as long as 3-6 weeks after a radical cure of infection [9-11] and possibly even longer if molecular detection tools are used for gametocyte detection. Microscopy is notoriously insensitive for detecting low density gametocytes [6,12] while molecular assays are sufficiently sensitive to detect and quantify gametocytes at densities as low as 0.02-0.1 gametocytes per microlitre [4]. These submicroscopic gametocyte densities can lead to infections in mosquitoes [13,14]. The use of molecular techniques has revealed that 80-90% of symptomatic malaria cases may carry gametocytes at clinical presentation and that this gametocyte carriage may persist for several weeks after successful treatment [15,16]. There is, therefore, a need to revisit previous estimates of the mean circulation time of gametocytes and the duration of gametocyte carriage using molecular detection tools.

Understanding the duration of gametocyte carriage is particularly important for determining the role of gametocytocidal anti-malarial drugs in the elimination of malaria [3]. The impact of widely used artemisinin-based combination therapy (ACT) on gametocyte carriage is pronounced [17,18] but not fully understood. The effect of artemisinins may be restricted to immature gametocytes [3,19-21] and may even be incomplete against these stages [22]. However, two *in vitro *studies observed additional activity of artemisinins against mature gametocytes [20,21] although for one study this could be explained by incomplete gametocyte synchronization [20]. *In vivo *studies commonly observe that mature gametocytes can persist after ACT treatment and result in post-treatment malaria transmission [16,22-24]. Primaquine (PQ) may have a more pronounced effect against mature gametocytes [3,11,19,25] although detailed field data are scarce, particularly in Africa where reports are inconclusive about the added value of PQ in clearing gametocytes persisting after ACT [15,26].

Here, by fitting simple mathematical models to data from two field trials which used sensitive molecular gametocyte detection methods, the mean gametocyte circulation time was determined and the full duration of gametocyte carriage was estimated in patients treated with non-ACT and gametocytocidal ACT and ACT-PQ drug combinations.

## Methods

Data were used from two clinical trials. Characteristics of the studies are summarized in table 1. The first trial was conducted in 2003 and 2004 in Mbita, western Kenya where the entomologic inoculation rate (EIR) was ~70 infectious bites per person per year (ibpy)[27]. This trial compared sulphadoxine-pyrimethamine alone (SP: 25 mg/kg S + 1.25 mg/kg P as single dose) or in combination with artesunate (AS: 4 mg/kg once daily for 3 days) or amodiaquine (AQ: 10 mg/kg once daily for three days) with treatment with artemether lumefantrine (AL: 10 mg A + 60 mg L/5 kg twice daily for three days) [16,28]. The second trial was conducted in 2006 in Mnyuzi, northeastern Tanzania (EIR ~10 ibpy [29]) and compared treatment with SP+AS with SP+AS followed by a single dose of primaquine (PQ: 0.75 m/kg once on the last (third) day of treatment)[15,30]. Both trials had similar inclusion criteria, enrolling children with uncomplicated malaria with a *P. falciparum *infection at a density of 500 - 100,000 parasites/μL and no microscopical evidence of co-infection with other *Plasmodium *species. Two microscopists each read 100 high power microscopic fields for asexual parasites. The trial in Kenya included children aged 0.5-10 years and excluded children with an Hb < 5 g/dL; the trial in Tanzania included children aged 3-15 years and excluded children with an Hb < 8 g/dL. Parents or guardians provided written informed consent for enrolment and the trial protocols received ethical clearance in Kenya (Scientific Steering Committee and Ethical Review Committee of the Kenya Medical Research Institute; SSC No. 791), Tanzania (National Institute for Medical Research; NIMR/HQ/R.8a Vol. XIII/446) and the UK (London School of Hygiene and Tropical Medicine #4097).

**Table 1:** Baseline description of the two studies that provided data for the current analysis

	Kenya, 2003-2004	Tanzania, 2006
Number of participants	160*	108

Age, median (IQR)	3 (1-5)	5 (3-9)

Drugs, (n)	Non-ACT: SP+AQ (127) ACT: SP+AS (174) & AL (75)	ACT: SP+AS (54) ACT-PQ: SP+AS+PQ (54)

Enrolment asexual microscopic parasite density, geometric mean (95% CI)	11,813 (9,690 - 14,402)	7,440 (1,000 - 24,280)

Microscopic gametocyte prevalence at enrolment, % (n/N)	25.5 (40/157)	22.6 (24/106)

*Pfs*25 QT-NASBA gametocyte prevalence at enrolment, % (n/N)	Non-ACT: 91.1 (41/45): ACT: 89.3 (100/112)	ACT: 88.2 (45/51) ACT-PQ: 92.3 (48/52)

*Pfs*25 QT-NASBA gametocyte density at enrolment, geometric mean/μL (95% CI)	Non-ACT: 1.22 (0.58-2.56) ACT: 0.66 (0.42-1.03)	ACT: 20.26 (8.08-50.37) ACT-PQ: 9.90 (4.43-22.11)

Included in current model fitting analysis	Non-ACT: 36^¶^ ACT: 90^¶^	ACT: 36^¥^ ACT-PQ: 41^¥^

IQR = interquartile range; SP = Sulphadoxine-pyrimethamine; AQ = amodiaquine; AS = artesunate; AL = artemether-lumefantrine; PQ: primaquine. * SP treated individuals (n = 152) or those without QT-NASBA data (n = 216) were excluded from the current analyses; ^¶^34 individuals were excluded because of treatment failure (n = 28) and/or failure to develop gametocytes during follow-up (n = 6); ^¥^26 individuals were excluded because of treatment failure (n = 21) and/or failure to develop gametocytes during follow-up (n = 8) and/or missing data (3).

### Gametocyte detection

Gametocyte carriage was determined on day 0, 3, 7, 14, 28 after treatment; treatment (d0-2) was completed 24 hours before the second time-point for gametocyte detection. For the trial in Tanzania, gametocyte carriage was also determined on day 42. In both studies, gametocyte prevalence and density were determined by examining 100 microscopic fields specifically for gametocytes and by quantitative nucleic acid sequence based amplification (QT-NASBA). The latter method is based on the detection of *Pfs25 *mRNA that is expressed in mature gametocyte (stage V) gametocytes [31] and has a sensitivity of 0.02-0.1 gametocyte per μL [32]. Enrolment gametocyte prevalence in the different treatment arms was 19-26% by microscopy and 85-92% by QT-NASBA [15,16].

### Data analysis

This study aimed to determine the mean circulation time of gametocytes and the impact of ACT and PQ on existing gametocyte carriage. Therefore, individuals were selected who i) had gametocytes by *Pfs*25 QT-NASBA at any time between day 0 and day 28 after enrolment, ii) were successfully cured of their asexual parasites (defined as no asexual parasites after day 3 and a day 3 asexual density≤10% of the day 0 density) and had no detectable re-infection. SP-treated children experienced a very high treatment failure rate by microscopy (56%). Additional low level SP-treatment failures may have been missed by day 28 [33] and the slow parasite clearance time probably extended the generation of gametocytes from asexual stages after the initiation of SP treatment [34]. The SP monotherapy arm was therefore completely excluded from the current analyses. Instead, models were fitted on data from i) SP+AQ treated individuals (non-ACT arm) since this drug combination had a high efficacy and rapid asexual parasite clearance time [16,35] but no activity against mature gametocytes[5]; ii) SP+AS and AL treated individuals that were combined as the ACT-arm since both drug combinations had an indistinguishable impact on gametocyte carriage [16]; iii) SP+AS+PQ (ACT-PQ) treated children. Frequent re-infections rendered the data from the trial in Tanzania less reliable after day 28 [15] and, therefore, only data up to this time-point were considered. Two phases in gametocyte carriage after treatment were considered. The first phase comprises day 0-3 when short acting artemisinins [36] and PQ [37,38] may have a direct effect on gametocyte densities and gametocyte densities may be influenced by an efflux of sequestered gametocytes as a result of the drug treatment induced 'stress' [7]. The initial reduction in gametocyte carriage in this period was quantified by expressing the gametocyte density on day 3 as a proportion of the enrolment density for the three treatment arms. The second phase was defined as day 3 till day 28 (end of follow-up) and was used to estimate the mean circulation time of gametocytes after asexual parasites have been cleared and gametocytocidal drug levels have waned.

### Mathematical model

**Figure 1:**
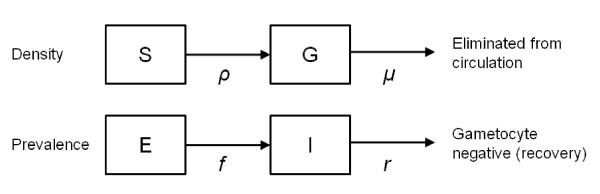
**Models describing change in gametocyte density and gametocyte prevalence over time**. S, sequestered gametocytes; G, circulating gametocytes; E, gametocyte-negative infected individuals (i.e. gametocytes not yet released into circulation); I, gametocyte-positive individuals; *ρ *rate of release of gametocytes from sequestration into the bloodstream; *μ*, rate of decay/removal of gametocytes; *f*, rate at which gametocyte-negative individuals become gametocyte-positive; *r*, rate at which gametocyte-positive individuals become gametocyte negative.

A simple deterministic compartmental model describing the gametocyte density per μL blood over time (Figure 1) was fitted to data on gametocyte density to estimate the circulation time per gametocyte. A second similar model with a prevalence framework rather than a density framework, describing the appearance of gametocytes in the blood (if not already present on day 0) and the time until they were fully cleared from the blood, was fitted to gametocyte prevalence data, to estimate the mean overall duration of gametocyte carriage in a patient following treatment (Figure 1). The structure of these models was similar to those of Hogh *et al *[39]. Both models assumed no further generation of gametocytes from asexual stages once treatment had been initiated; however, gametocytes were assumed to emerge from sequestration into the peripheral blood. The two models were fitted separately to data from individuals grouped according to treatment type into three previously described groups: non-ACT, ACT and ACT-PQ.

In the density model, the gametocyte concentration in the bloodstream *G *changes over time depending on the rate of release of gametocytes into the bloodstream *ρ *from a sequestered gametocyte population *S*, and on the decay rate *μ *of the circulating gametocytes. Exponentially distributed sequestration and decay times were assumed, so that 1/*ρ *gives the mean sequestration time and 1/*μ *is the mean circulation time per gametocyte. The differential equations describing this process are given by:

This is solved to obtain an expression for the expected gametocyte density in circulation at time t since treatment began, G(t), where *S_0 _*is the density of the sequestered gametocyte population on day 0, and *G_0 _*is the density of circulating gametocytes on day 0:

The model was fitted to the log gametocyte densities using maximum likelihood methods, assuming a Normal distribution for the log transformed data and incorporating Normally-distributed random effects for each patient in overall density. Zero density values in the data were treated as values below the detection limit of the assays (0.02 gametocytes/μL for the QT-NASBA method) and the likelihood was specified to account for these censored observations. Each trial was fitted separately as there was a significant difference in initial log gametocyte density between trials and therefore other factors may also have differed between the two study populations. Gametocyte circulation time was allowed to vary by treatment type, since both ACT and PQ may have differing effects. The initial size of the sequestered gametocyte population was an uncertain parameter. As well as estimating this parameter from the model fitting, a sensitivity analysis was undertaken for a range of fixed values. Because ACT is likely to clear immature gametocytes before they can be released into sequestration, we fixed the sequestered gametocyte population to be 3 log lower than for the non-ACT treatment (Table 2). The rate of release of sequestered gametocytes, and any differences between treatment groups in the initial size of the sequestered gametocyte population could not be estimated independently from other parameters but was assumed to be similar between treatment groups within trials. Different fixed values were therefore fitted for the size of the sequestered gametocyte population that was subsequently released into the circulation in a sensitivity analysis (Additional file 1). The duration of the sequestration period per gametocyte was previously determined at 4-12 days [9]; we fitted different values within this range and fixed the sequestration time to 11 days based on the best fit (Table 2).

**Table 2:** Other parameters used or estimated in density and prevalence models using QT-NASBA data

Parameter	Description	Estimates (95% CI)	
		**Kenya, 2003-2004**	**Tanzania, 2006**

**Density model**			

*1/ρ*	1/duration of gametocyte sequestration, days	1/11	1/11

S_3_*	size of sequestered gametocyte population on day 3 that is subsequently released into the circulation	Non-ACT: 0.0263 ACT: 0.0013	0.0013 (0.0001-0.0237)

*G_3_*^¶^	density of circulating gametocytes/μL on day 3 after start treatment	non-ACT: 1.45 (0.44-4.74) ACT: 0.13 (0.03-0.52)	ACT: 1.88 (0.37-9.52) ACT-PQ: 0.07 (0.01-0.86)

**Prevalence model**			

*1/f *^¥^	time until patients gametocyte-negative on day 0 become gametocyte-positive, days	5.60	5.60 (1.06-29.4)

All values with confidence intervals were estimated in the model; values without confidence intervals were fixed unless stated otherwise.

* estimated based on the trial data, expressed as the total sequestered population that would be released per μL of blood. Details of the sensitivity analysis for this parameter are given in table S1. The value presented here is a product of the baseline sequestered gametocyte density and the immediate impact of anti-malarial drugs on days 0, 1 and 2.

^¶^estimated based on the data for each trial and treatment arm separately. The density of circulating gametocytes at enrolment was not different between arms within trials (table 1). The value presented here is a product of the baseline gametocyte density and the immediate impact of anti-malarial drugs on days 0, 1 and 2.

^¥^Estimated from the data in the trial in Kenya. The number of children who became gametocytaemic during the trial while being gametocyte negative at enrolment was 3 (out of 4 gametocyte negative individuals) for non-ACT in Kenya, 9 (out of 12) for ACT in Kenya, 2 (out of 6) for ACT in Tanzania and 0 (out of 4) for ACT-PQ in Tanzania.

The gametocyte prevalence model describes the appearance and disappearance of any circulating gametocytes according to the prevalence at day 0, *I_0_*, the rate *f *at which initially gametocyte-negative proportion *E *move to the gametocyte-positive state *I*, and the rate *r *at which infectious individuals recover to become gametocyte negative. The equations describing this process are given by:

This can be solved to obtain an expression for the expected prevalence of gametocytes in the patients *t *days after treatment, *I(t)*, as:

The model was fitted to the individual patient prevalence data using maximum likelihood methods assuming a Binomial outcome distribution and random patient effects Normally distributed on the logit scale. The rate of loss of gametocytaemia, r, was allowed to vary by treatment type, while the other parameters were assumed to be constant between groups. The rate of becoming gametocyte positive, *f*, was estimated from the data, and also varied in sensitivity analysis [40]. The time to positivity and the duration of infectiousness were assumed to be exponentially distributed with means given by 1/*f *and 1/*r*, respectively. To obtain the predicted marginal probabilities of gametocyte positivity at each time point for each treatment group from estimated parameters, 10,000 values were simulated from the random effects distribution using the estimated variance from the model, added to the logit transformed model prediction, then transformed back to the prevalence scale.

All model fitting was carried out using the PROC NLMIXED procedure in SAS (Version 9.1, SAS Institute Inc, USA).

## Results

The numbers and characteristics of individuals included in the analysis are summarised in table 1. While there were no statistically significant differences in asexual parasite density or gametocyte prevalence or density between treatment arms within the trials [15,16], baseline values differed between trials. The density of asexual parasites was higher and the gametocyte density was lower in the trial in Kenya compared to the trial in Tanzania.

### The circulation time of gametocytes

**Figure 2:**
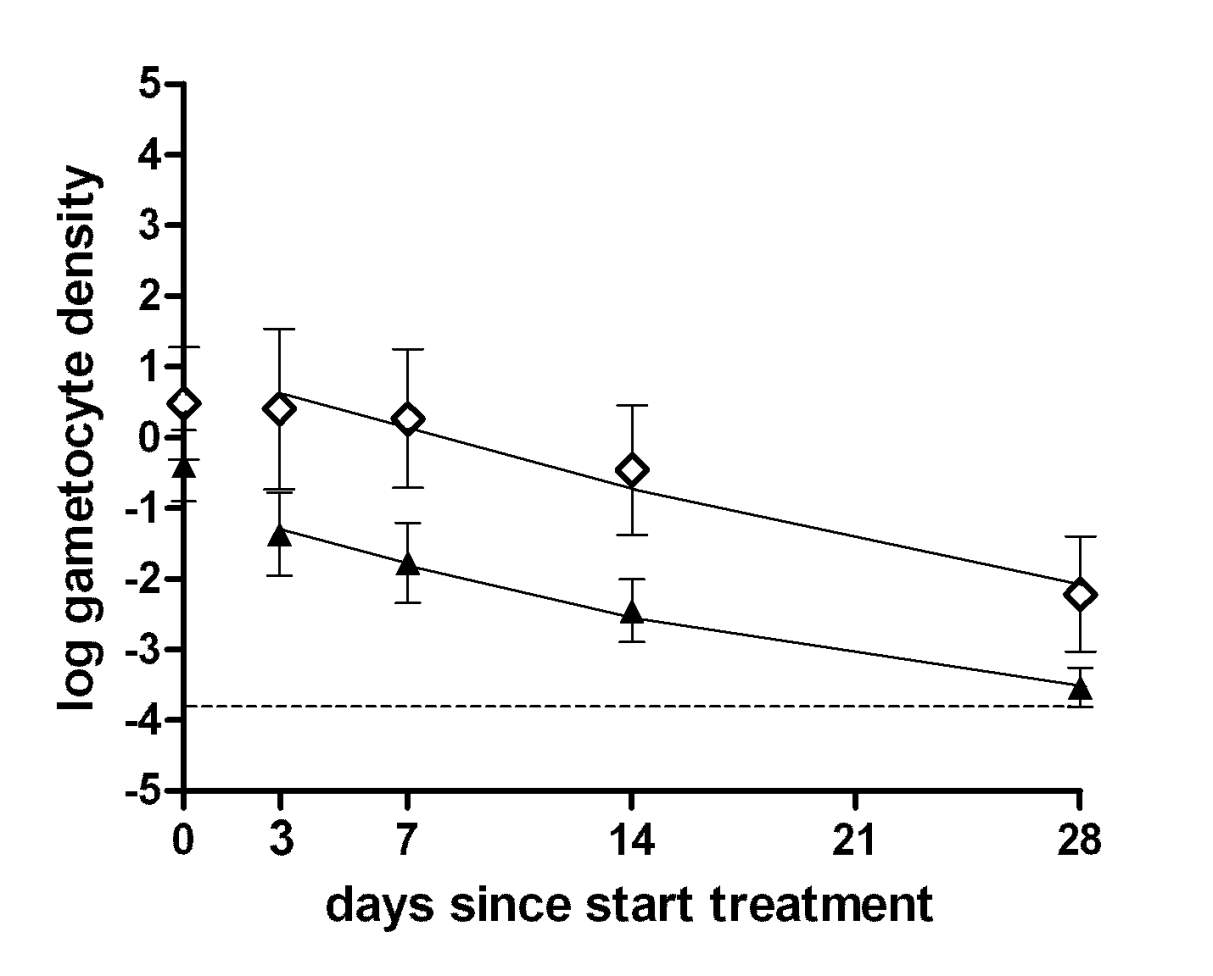
**The mean gametocyte circulation time based on *Pfs25 *QT-NASBA gametocyte density after non-ACT and ACT**. Log gametocyte density/μL is given on the Y-axis, the day of follow up after initiation of treatment. Symbols and error bars indicate the field data with 95% confidence interval, lines fitted values. The dotted line indicates the lower threshold for gametocyte detection by QT-NASBA, 0.02 gametocytes/μL. The trial was conducted in Kenya; treatment regimens were non-ACT (open diamonds; SP+AQ administered on day 0-2) and ACT (closed triangles; SP+AS or AL administered on day 0-2). The estimated mean circulation time of gametocytes in this trial was 6.53 days (95% CI 4.84-8.80) after non-ACT treatment and 5.04 days (95% CI 4.20-6.06) after ACT treatment, based on data between d3 and d28.

**Figure 3:**
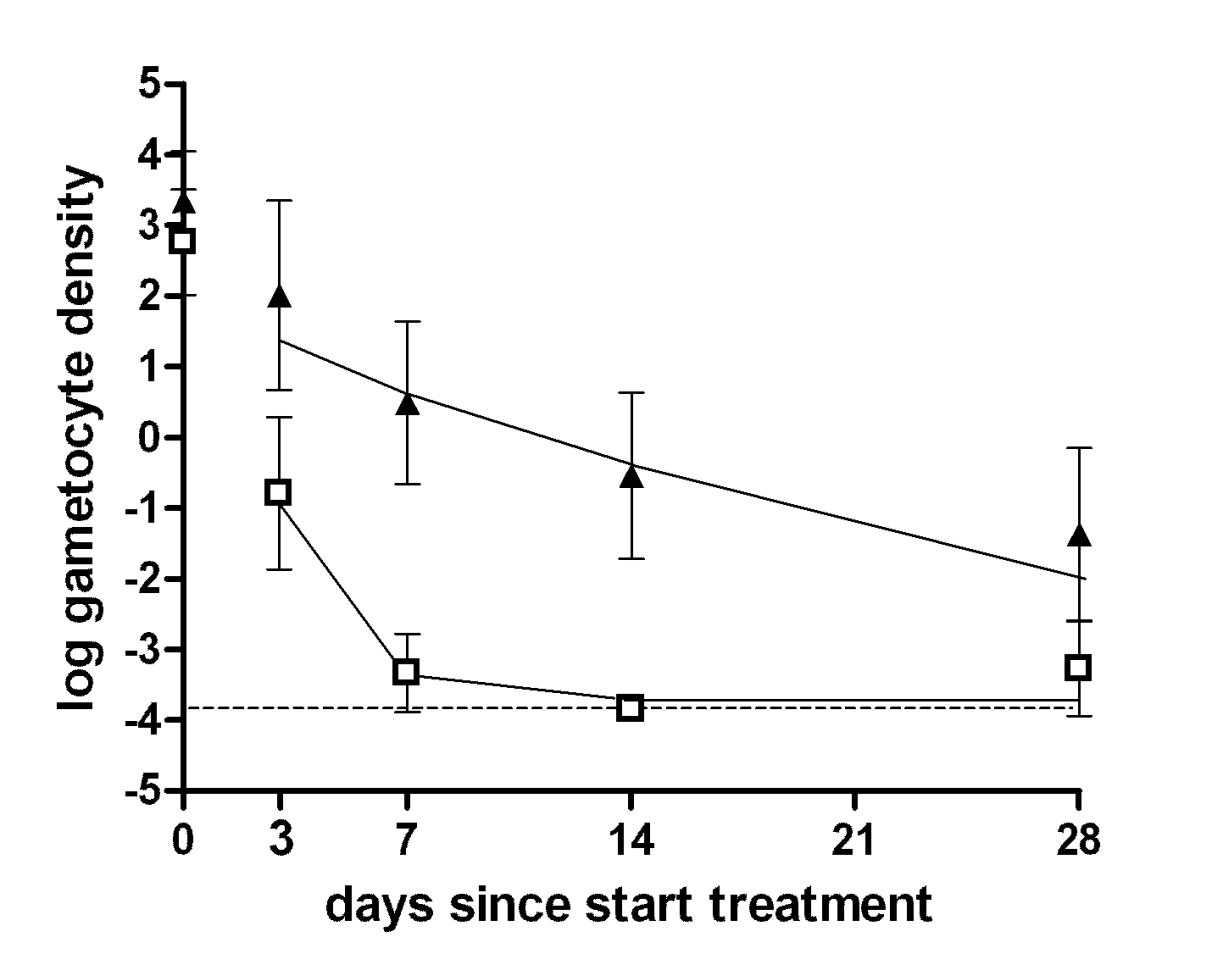
**The mean gametocyte circulation time based on *Pfs25 *QT-NASBA gametocyte density after ACT and ACT-PQ**. Log gametocyte density/μL is given on the Y-axis, the day of follow up after initiation of treatment. Symbols and error bars indicate the field data with 95% confidence interval, lines fitted values. The dotted line indicates the lower threshold for gametocyte detection by QT-NASBA, 0.02 gametocytes/μL. The trial was conducted in Tanzania; treatment regimens were ACT (closed triangles; SP+AS administered on day 0-2) and ACT-PQ (open squares; SP+AS administered on day 0-2 followed by a single dose of PQ on day 2). The mean circulation time of gametocytes in this trial was 4.61 days (2.92 - 7.26) after ACT treatment and 0.53 days (0.24-1.19) after ACT-PQ treatment.

The mean circulation time of gametocytes was estimated using *Pfs*25 QT-NASBA gametocyte density data. The data indicated an approximately linear decrease in log-gametocyte density between day 3 and day 28 (Figure 2), except in the ACT-PQ group where the gametocyte density rapidly approached zero (Figure 3). This gradual decline in gametocyte density was interpreted as the natural decay of gametocytes after peak gametocytocidal drug levels had waned [41]. When this time-frame (day 3-28) was used to fit the natural decay rate of gametocytes in the Kenyan trial, the mean gametocyte circulation time was estimated at 6.5 days (95% CI 4.8-8.8) and 5.0 days (95% CI 4.2-6.1) in the non-ACT and ACT treated individuals, respectively, with no statistically significant difference between the groups (p = 0.144). Using the same methodology, the mean gametocyte circulation time was 4.6 days (95% CI 2.9-7.3) after ACT in the Tanzanian trial (Figure 3). This estimate was lower than that of the trial in Kenya, although the difference was not statistically significant (p = 0.670). The gametocyte circulation time of patients treated with ACT-PQ was estimated to be significantly lower at 0.5 days (95% CI 0.2-1.2) (p < 0.001).

### The impact of drugs on gametocyte carriage

Prior to the gradual decline of gametocyte densities between day 3 and day 28, there was a sharp reduction in gametocyte densities in the ACT arm in the Kenyan trial. In children in the ACT group who carried gametocytes at enrolment, the density of gametocytes fell by a median of 90.9% (IQR 39.0-100.0%; p < 0.001) between day 0 and day 3. In the Tanzanian trial, the median reduction in gametocyte density between day 0 and day 3 after initiation of ACT treatment was 67.7% (IQR 33.5-95.3%, p < 0.001). There was no statistically significant change in gametocyte density in the first three days after initiation of efficacious non-ACT treatment (p = 0.10). The ACT-PQ combination had a stronger and longer impact on gametocyte carriage than ACT alone. The gametocyte density decreased very sharply between day 0 and day 3 (median reduction 99.1%, IQR 71.8-100%) and, rather than a gradual decline, continued to decrease sharply afterwards. Between day 3 and day 7, gametocytes were cleared for 76.9% (20/26) of the children who still had gametocytes by day 3. Only 4.9% (2/41) of all gametocytaemic children at enrolment still had detectable levels of gametocytes by day 14 after ACT-PQ treatment. Between day 14 and day 28, one of these two individuals remained gametocytaemic. Four others became gametocytaemic after being gametocyte free on day 3, 7 and 14 (n = 3) or on day 7 and 14 (n = 1), suggesting that their gametocytes were produced by a newly acquired infection or parasites that emerged from the liver or recrudesced after treatment was initiated.

**Figure 4:**
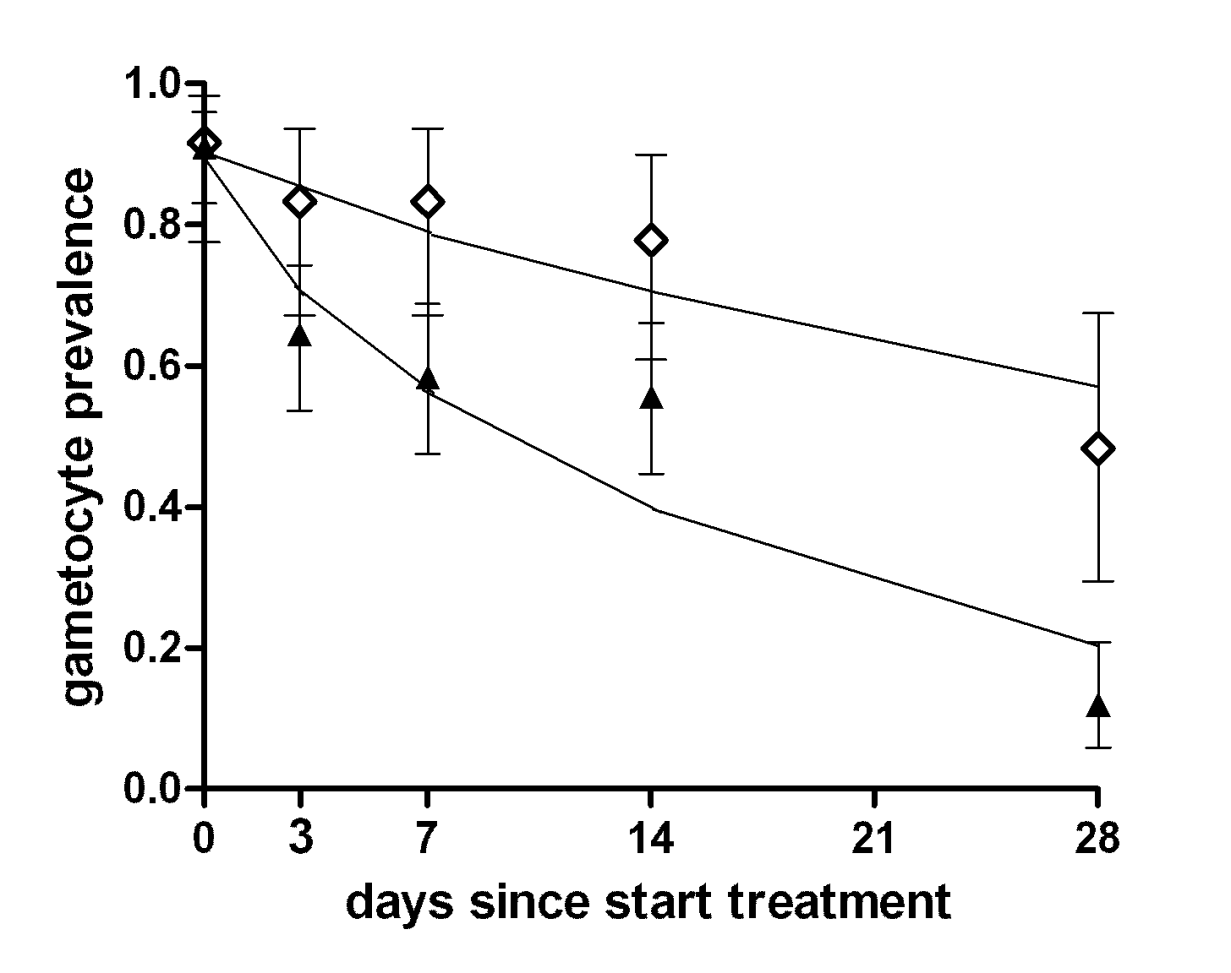
**The duration of gametocyte carriage based on the *Pfs25 *QT-NASBA gametocyte prevalence after non-ACT and ACT treatment**. Symbols and error bars indicate the field data with 95% confidence interval, lines fitted values. The trial was conducted in Kenya; treatment regimens were non-ACT (open diamonds; SP+AQ administered on day 0-2) and ACT (closed triangles; SP+AS or AL administered on day 0-2). The estimated average duration of gametocyte carriage was 55.6 days (95% CI 28.7-107.7) after non-ACT treatment and 13.4 days (95% CI 10.2-17.5) after ACT treatment.

**Figure 5:**
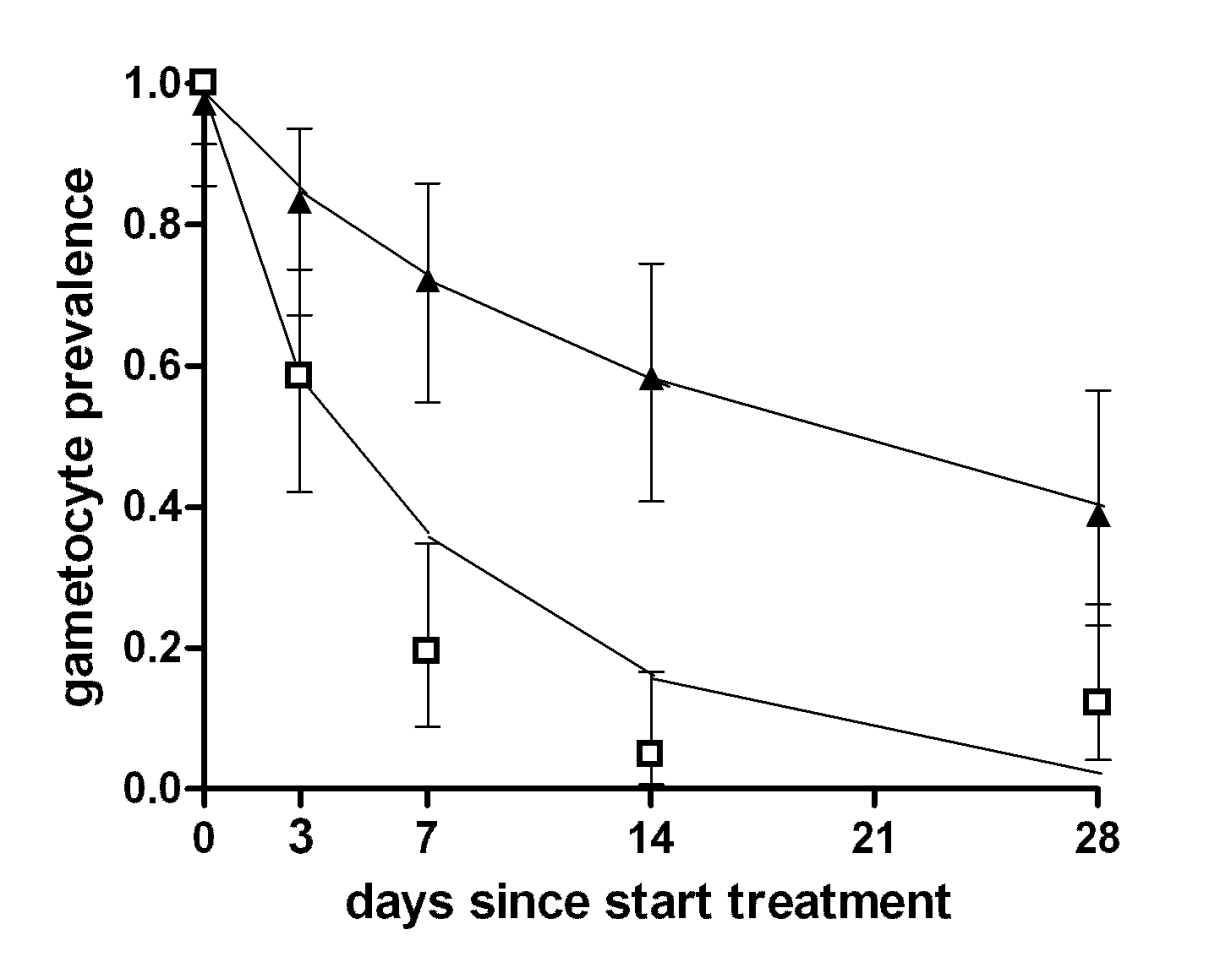
**The duration of gametocyte carriage based on the *Pfs25 *QT-NASBA gametocyte prevalence after ACT and ACT-PQ treatment**. Symbols and error bars indicate the field data with 95% confidence interval, lines fitted values. The trial was conducted in Tanzania; treatment regimens were ACT (closed triangles; SP+AS administered on day 0-2) and ACT-PQ (open squares; SP+AS administered on day 0-2 followed by a single dose of PQ on day 2). The estimated average duration of gametocyte carriage was 28.6 days (95% CI 17.0 - 48.0) after ACT treatment and 6.3 days (95% CI 4.7-8.5) after ACT-PQ treatment.

The variation between study drugs and trials was reflected in the estimated duration of gametocyte carriage after treatment (Table 3). It was necessary to estimate the duration of gametocyte carriage with a model because it was beyond the last time point of follow-up for some drugs. In the trial in Kenya, the mean duration of gametocyte carriage was estimated to be 55.6 (95% CI 28.7 - 107.7) days after successful non-ACT treatment; 48.3 (14/29) of the children still harboured gametocytes on day 28 after non-ACT treatment (Figure 4). In the same trial, the use of ACT resulted in a four-fold reduction of the estimated duration of gametocyte carriage compared to non-ACT (13.4 days; 95% CI 10.2-17.5). The duration of gametocyte carriage after ACT treatment was longer in the trial in Tanzania compared to the trial in Kenya (28.6 days; 95% CI 17.0 - 48.0; p = 0.011). Addition of PQ to ACT resulted in a marked fourfold reduction in the duration of gametocyte carriage to 6.3 (95% CI 4.7-8.5) days (Figure 5).

**Table 3:** The duration of gametocyte carriage and persistence of gametocytes after treatment with non-ACT, ACT and ACT-PQ in clinical trials in Kenya and Tanzania.

	Duration of gametocyte carriage in days (95% CI)	% still gametocytaemic during follow up (n/N)	
		**Day 14**	**Day 28**

**Kenya, 2003-2004**			

Non-ACT	55.6 (28.7-107.7)	77.8 (28/36)	48.3 (14/29)

ACT	13.4 (10.2-17.5)	55.6 (50/90)	11.9 (10/84)

p-value	< 0.001	0.02	< 0.001

**Tanzania, 2006**			

ACT	28.6 (17.0-48.0)	58.3% (21/36)	38.9% (14/36)

ACT-PQ	6.3 (4.7-8.5)	4.9%(2/41)	12.2% (5/41)

p-value	< 0.001	< 0.001	0.007

All individuals included in the analyses had gametocytes by *Pfs25 *QT-NASBA at enrolment and were successfully treated, i.e. were free of microscopic asexual parasitaemia between day 3 and the end of follow-up.

## Discussion

In the current study, previous estimates of the circulation time of *P. falciparum *gametocytes were revisited using sensitive molecular gametocyte detection methods. The mean circulation time of gametocytes was 4.6-6.5 days, depending on the study site. Gametocyte carriage persisted for an estimated average of 55 days after the initiation of treatment that resulted in the apparent clearance of asexual parasites. ACT shortened the period of gametocyte carriage considerably, particularly when combined with PQ, a regimen that had the most pronounced effect on mature gametocytes.

Findings on circulation time of gametocytes confirm previous estimates that used microscopy for gametocyte detection. Although the molecular gametocyte detection method that was used was considerably more sensitive for detecting low-density gametocyte carriage [4,6], the rate of decline of gametocyte density over time was similar to that observed by microscopy [8,9]. Not surprisingly however, the higher sensitivity for gametocyte detection did result in a longer estimated duration of gametocyte carriage after successful treatment. The duration of gametocyte carriage after treatment depends on the maximum sequestration time and the maximum circulation time. The average gametocyte circulation time in this study was 4.6-6.5 days, but the maximum circulation time will considerably longer and was previously estimated at 22.2 days [9]. The field data presented in this study indicate that approximately half of the children still carried gametocytes four weeks after the initiation of efficacious non-ACT treatment. Fitting a mathematical model to these data, the average duration of gametocyte carriage was 55 days after initiation of treatment. This estimate was obtained by extrapolating the gradual decline in gametocyte prevalence observed between day 0 and day 28 to later time-points, thereby assuming that the pattern of loss follows an exponential distribution at a rate that does not change with time. If however, the rate of decay increases after day 28 the true duration of gametocyte carriage would be lower than reported here. The duration of gametocyte carriage can therefore not be estimated with certainty after the last day of follow-up, day 28. The sampling scheme was based on conventional time-points for drug sensitivity trials and extending the follow-up to several months would have been logistically challenging and introduced a high chance of enumerating gametocytes derived from re-infections. The estimate of 55 days is also longer than the sum of previously reported maximum sequestration and maximum circulation times, which would be 32 days [9]. The findings nevertheless demonstrate that gametocyte carriage can persist for well beyond one month after the apparent clearance of asexual parasites and in some individuals gametocyte carriage after efficacious non-ACT treatment may persist for longer than previous anecdotal reports of 3-6 weeks [9-11]. Although gametocyte densities and the associated likelihood of malaria transmission to mosquitoes will decrease with time, individuals may remain infectious to mosquitoes for several weeks after successful clearance of asexual parasites [13]. This highlights the importance of the use of gametocytocidal drugs to reduce the duration of gametocyte carriage.

While the non-ACT combination had no activity against mature gametocytes [5], both ACT and ACT-PQ had a pronounced effect on the density of circulating gametocytes. In the trial comparing non-ACT with ACT treatment, ACT reduced the duration of gametocyte carriage fourfold. This was solely explained by the impact of ACT on gametocytes in the first days of the study. After day 3, when artemisinin concentrations are ineffective [36], a gradual decline of gametocyte densities over time was observed that was not different from that in non-ACT treatment and interpreted as the natural decay of gametocytes. This explains why the mean gametocyte circulation time after day 3 is similar between non-ACT and ACT while the duration of gametocyte carriage is very different. The latter is influenced considerably by the early effects of treatment, i.e. the reduction in gametocyte density between day 0 and day 3. The *Pfs*25 mRNA-based detection method only detects mature stage V gametocytes [31]. Assuming a complete clearance of asexual parasites, the post-treatment concentration of mature gametocytes in the circulation is influenced by i) the circulating gametocyte density at enrolment, ii) the release of sequestered gametocytes upon treatment [7], iii) the natural death rate of gametocytes, iv) possible immune clearance of gametocytes [42] and v) the activity of anti-malarials against mature gametocytes. The difference between non-ACT and ACT in the early reduction in gametocyte densities is at least partly explained by the clearance of immature sequestered gametocytes that are released into the circulation at a much lower rate after ACT [3,22]. While the concentration of mature gametocytes remained unaltered shortly after efficacious non-ACT (i.e. the release of sequestrated gametocytes and decay of circulating gametocytes are in balance), the gametocyte concentration was 65-90% lower after three days of ACT. It is unclear whether this is (partly) the result of an effect against mature gametocytes [21] or solely of a rapid clearance of sequestered immature gametocytes. One population averaged value was fitted for the pool of sequestered gametocytes that was released into the circulation after treatment. The best estimate from the model was that this pool comprised less than 0.01% of the mean circulating pool of gametocytes. A sensitivity analysis indicated that the exact value of this parameter did not change the estimated circulation time of gametocytes after treatment substantially unless values of >15% of circulating gametocytes were used. The uncertainty of this parameter is a shortcoming of the presented model and a challenge for future assays for testing gametocytocidal drugs. Ideally, one would directly determine the sequestered gametocyte pool for each individual to allow for influences of peripheral blood gametocyte density and the duration of infection. For asexual *P. falciparum *parasites, it is possible to estimate the circulating and sequestered number of parasites by measuring plasma concentration of histidine-rich protein (PfHRP2) [43]. A similar approach is currently unavailable for gametocytes, but would greatly facilitate the assessment of the activity of anti-malarial drugs against different stages of *P. falciparum *gametocytes.

Addition of PQ to ACT was found to strongly increase the clearance of remaining gametocytes [15]. A recently published mathematical model indeed suggests that malaria transmission may be significantly lowered in a low transmission setting by PQ in addition to ACT [38]. Here, ACT-PQ resulted in a four-fold reduction of the estimated duration of gametocyte carriage compared to ACT alone. This could be partly explained by PQ clearing liver stage infections, thereby preventing the production of gametocytes from parasites newly released from the liver. However, the rapid decline in mature gametocytes upon treatment indicates that the activity against circulating gametocytes will be most important. In eight individuals (8/41, 19.5%) low densities of gametocytes persisted until the fifth day after PQ administration (day 7 after initiation of treatment) and in two individuals (2/41, 4.9%) gametocytes persisted until the twelfth day after PQ administration (day 14 after initiation of treatment). PQ concentrations peak approximately two hours after administration [37] and PQ has an elimination half-life of approximately 8-hours [37,38]. This raises questions about the nature of the apparent reduction of gametocyte densities several days after PQ treatment. Previous studies also reported that microscopically detectable gametocytes were detectable for several days after PQ administration before they were eventually cleared [19,44]. The exact mechanism by which PQ clears gametocytes is unknown but may be related to an effect of PQ on mitochondrial proliferation that inhibits development stages, such as gametocytes, that require functional mitochondria [45]. The cause of the observed 'delayed effect' of PQ is unclear but exposure to (the active metabolites of) PQ appears to influence the longevity of mature gametocytes that initially survive peak plasma concentrations. This is reflected in a reduced estimated circulation time per gametocyte between days 3 and 28 compared to ACT or non-ACT treatment.

The presented analysis has several limitations in addition to the fact it is based on only two studies. The confounding potential of re-infections was minimised by limiting the observation period to a time when re-infection was deemed unlikely. However, it is possible that some low-density re-infections or persisting parasitaemia occurred, as was suggested by the increase in gametocyte prevalence in the trial in Tanzania after gametocytes were cleared from all but one individual. There is currently no molecular detection tool that specifically detects asexual parasites with the same sensitivity as the gametocyte *Pfs*25 QT-NASBA. Undetected low density infections could have boosted gametocyte production [7] or resulted in ongoing gametocyte production [46] and resulted in a longer estimated duration of gametocyte carriage. It is also impossible to exclude that parasites newly emerging from the liver have contributed to gametocyte production after drug levels had waned. This does not affect the validity of the conclusions, i.e. gametocyte carriage can persist for several weeks after treatment, but underlines the importance of developing new molecular parasite detection tools to confirm the origin of post-treatment gametocytes. These tools should be sensitive, quantitative and stage specific. The differences between study sites also highlight the limitations in extrapolating findings from one study area. Gametocyte dynamics may differ between areas [6] and the findings indicate that this may also influence the impact of treatment on gametocyte carriage. The duration of gametocyte carriage appeared longer in the Tanzanian trial (28.6 days compared to 13.4 days in Kenya). Enrolment *Pfs*25 QT-NASBA gametocyte densities were higher in the Tanzanian trial, which may explain the observed difference, given that ACT is thought to impact less on mature gametocytes. Increased clonal complexity [47] and younger age [10] have both been associated with a longer duration of gametocyte carriage. However, these factors cannot explain the observed difference between trials since transmission intensity is positively correlated with multiplicity of infection [48], but lower at the Tanzanian site [29], where trial participants were also older.

## Conclusion

The current analysis indicates that gametocytes persist for an average of more than one month after clearance of asexual parasites. Artemisinins can shorten the duration of gametocyte carriage approximately fourfold. PQ is a more potent gametocytocidal drug than artemisinins and in led to a fourfold reduction in the duration of gametocyte carriage compared to ACT alone.

## Conflicts of interests

The authors declare that they have no competing interests.

## Authors' contributions

TB and LO analysed the data and wrote the manuscript; TB, SS, SO, PS and RS were responsible for the original study design and data collection; JTG and ACG contributed in data analysis; CS, RS, ACG and CD contributed to data interpretation and manuscript preparation. All authors read and approved the final manuscript.

## Supplementary Material

Additional file 1 **Circulation time of gametocytes: sensitivity of model fits to assumed size of sequestered gametocyte population at day 0 (S_0_)**. This table contains the outcomes of a sensitivity analysis where different fixed values of the sequestered gametocyte population were fitted. The impact on the estimated circulation time of gametocytes is presented for each trial and treatment arm. Click here for file
