# Supplementary material for: Revisiting the circulation time of Plasmodium falciparum gametocytes: molecular detection methods to estimate the duration of gametocyte carriage and the effect of gametocytocidal drugs
Source: Malar J. 2010 May 24;9:136. doi: 10.1186/1475-2875-9-136 (PMC2881938; doi:10.1186/1475-2875-9-136)
Supplement: Additional file 1 — Circulation time of gametocytes: sensitivity of model fits to assumed size of sequestered gametocyte population at day 0 (S0). This table contains the outcomes of a sensitivity analysis where different fixed values of the sequestered gametocyte population were fitted. The impact on the estimated circulation time of gametocytes is presented for each trial and treatment arm. [file 1475-2875-9-136-S1.DOC]

**Additional file 1. Circulation time of gametocytes: sensitivity of model fits to assumed size of sequestered gametocyte population at day 0 (S_0_**).

|  |  | **Circulation time of gametocytes** | | | |
| --- | --- | --- | --- | --- | --- |
|  |  | **Kenya, 2003-2004** | | **Tanzania, 2006** | |
| S_0_ Non- ACT (log) | S_0_ ACT (log) | non ACT | ACT | ACT | ACT-PQ |
| 2.7183 (1) | 0.1353 (-2) | 2.63 (1.13-6.09) | 2.95 (1.97-4.41) | 3.18 (1.47-6.9) | 0.01 (0-0.06) |
| 1.0000 (0) | 0.0498 (-3) | 4.32 (2.68-6.96) | 3.86 (3.03-4.91) | 4.1 (2.36-7.12) | 0.018 (0.0022-0.16) |
| 0.1353 (-2) | 0.0067 (-5) | 6.03 (4.3-8.44) | 4.54 (3.72-5.55) | 4.56 (2.87-7.22) | 0.14 (0.02-1.12) |
| 0.0263 (-3.64)^*^ | 0.0013 (-6.64)^*^ | 6.53 (4.84-8.8) | 5.04 (4.2-6.06) | 4.61 (2.92-7.26) | 0.53 (0.24-1.19) |
| 0.0009 (-7) | 0.0000 (-10) | 6.7 (4.9-9.17) | 4.85 (4.03-5.83) | 4.42 (2.76-7.09) | 0.61 (0.36-1.02) |
| 0.0000 (-10) | 0.0000 (-13) | 6.71 (4.91-9.17) | 4.85 (4.03-5.83) | 4.51 (2.81-7.26) | 3.35 (2-5.59) |

^*^best fitted value
